# Solitary Extramedullary Plasmacytoma With Development of T-cell Anaplastic Large-Cell Lymphoma: A Rare Case Report and Literature Review

**DOI:** 10.7759/cureus.38153

**Published:** 2023-04-26

**Authors:** Maria Voronova, Po-Hua Chen, Artem Sharko

**Affiliations:** 1 Internal Medicine, I.M. Sechenov First Moscow State Medical University, Moscow, RUS; 2 Internal Medicine, Chicago Medical School, Rosalind Franklin University of Medicine and Science, North Chicago, USA; 3 Internal Medicine, Northwestern Medicine McHenry Hospital, McHenry, USA

**Keywords:** plasmacytoma treatment, gastrointestinal oncology, solitary extramedullary plasmacytoma, lymphoma, plasmacytoma

## Abstract

Solitary extramedullary plasmacytomas are an exceedingly rare form of malignant neoplasms characterized by a single localized mass located in any soft tissue that consists of abnormal plasma cells. This type of tumor is characterized by the absence of plasmacytosis on bone marrow biopsy, the absence of other lesions on imaging, and no clinical signs of multiple myeloma. They usually present with mass effect, so the clinical picture varies based on the location of the tumor. In cases where the tumor is located in the gastrointestinal tract, patients may experience abdominal pain, small bowel obstruction, or gastrointestinal bleeding. The diagnostic process typically involves imaging to identify the tumor and its location, followed by a biopsy of the lesion with subsequent immunohistochemical analysis, as well as fluorescence in situ hybridization, and finally, bone marrow biopsy. Treatment options vary depending on the tumor's location and may include radiation therapy, surgical resection, and chemotherapy. Currently, radiation therapy is the preferred first-line treatment, with the best outcomes reported in the literature. Surgery is also frequently used and is often followed by radiation therapy. While chemotherapy has not been shown to have significant benefits, the available data is insufficient, and further studies are required to make better conclusions. Disease progression is often associated with transformation to multiple myeloma, but due to the rarity of the disease, data is limited, and it remains unclear if other forms of progression exist. We report a case of a 63-year-old male who presented to the hospital with symptoms of abdominal pain, nausea, and vomiting. A computed tomography scan revealed a mass causing bowel obstruction, which was subsequently resected and evaluated by pathology. The final diagnosis was determined to be a solitary extramedullary plasmacytoma. Since the margins of the resected mass were clear, the patient was managed with solely clinical observation. Approximately eight months later, the patient was diagnosed with T-cell anaplastic large-cell lymphoma, ultimately leading to his passing 15 months after the initial diagnosis of solitary extramedullary plasmacytoma. We present this case to increase awareness of the rare condition of solitary extramedullary plasmacytoma and to highlight the potential association with T-cell anaplastic large-cell lymphomas, as demonstrated in this patient's case. Given the possibility of malignant transformation, close monitoring is warranted in similar cases.

## Introduction

Solitary extramedullary plasmacytomas (SEP) are plasma cell neoplasms that arise in soft tissues without the involvement of the bone marrow (BM) and lack clinical signs of multiple myeloma (MM) [1]. They occur in approximately 0.1 of 100,000 individuals, with the most common location being in the head and neck soft tissues and upper respiratory tract, including the nasal cavity, sinuses, pharynx, and larynx [1,2]. SEP in the gastrointestinal (GI) tract is extremely rare, accounting for only 4% of cases [3]. Diagnosis typically involves imaging, a biopsy of the tumor, and a BM biopsy for further evaluation using immunohistochemical (IHC) analysis and fluorescence in situ hybridization (FISH) techniques [1,3,4]. Radiation therapy is the primary treatment option, with surgical resection being necessary in cases presenting as a mass effect. Chemotherapy has been used occasionally; however, data regarding its effectiveness is inconclusive [1,5]. Due to the rarity of SEP in the GI tract, data on management, prognosis, and disease progression is limited.

We present a case of a 63-year-old male with abdominal pain and small bowel obstruction who was ultimately diagnosed with SEP of the jejunum. Our aim is to increase awareness of this rare condition, share our experience in managing this patient, and highlight a potential variant of progression that has not been previously reported, the development of T-cell anaplastic large-cell lymphoma.

## Case presentation

**Figure 1:**
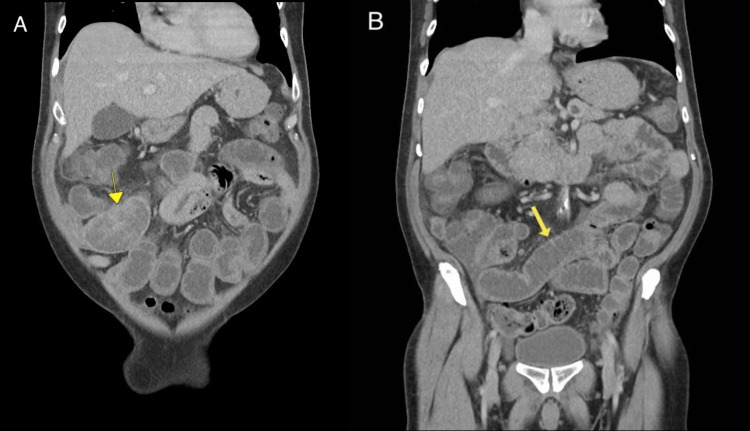
CT of the abdomen: A 2.2 x 4.9 cm mass (yellow arrow) is seen in the distal ileum (A), and the small bowel loops proximal to the mass appear dilated (B). CT - computed tomography

The patient is a 63-year-old male who presented to the emergency department (ED) with intermittent, colicky abdominal pain in the right lower quadrant associated with nausea, non-bloody vomiting, and an inability to pass stool or gas for about 48 hours. The patient denied blood in his stool or any other symptoms. Upon examination in the ED, the patient's vital signs were normal, and a physical exam revealed tenderness to palpation in the right lower quadrant of the abdomen. Lab results showed mild leukocytosis, but other tests were within normal limits, including hemoglobin, platelet, and complete metabolic panel (CMP). A computed tomography (CT) scan of the abdomen and pelvis showed a 2.2 x 4.9 cm heterogeneous mass in the distal ileum with small bowel obstruction and dilated small bowel loops measuring up to 3.3 cm in diameter proximal to the obstruction (Figure 1).

**Figure 2:**
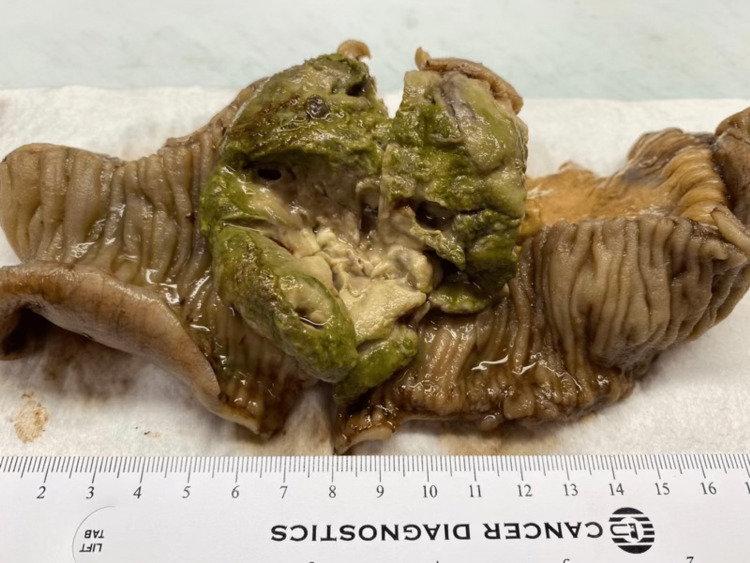
Pathology specimen: An 8.5 cm tumor involving the full thickness of the bowel wall is seen.

**Figure 3:**
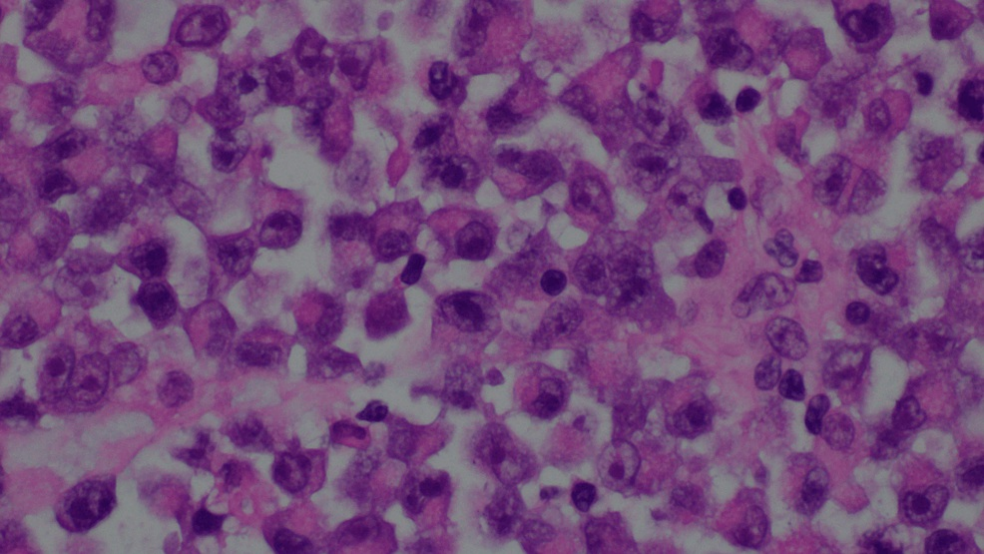
Hematoxylin and eosin stain: Round and discohesive tumor cells are present. Many display a prominent central nucleolus and pink cytoplasmic globules morphologically consistent with Russell and Dutcher bodies.

**Figure 4:**
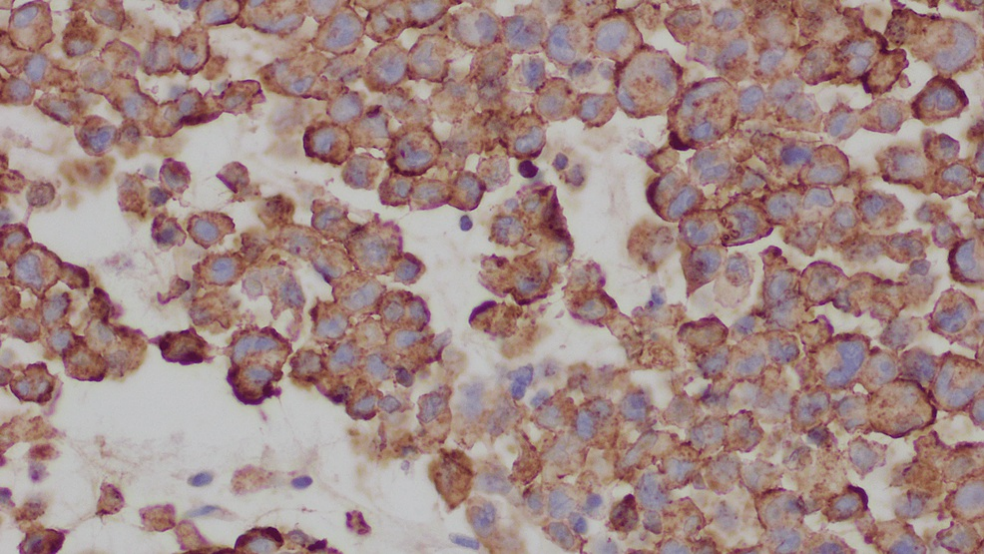
IHC analysis: Tumor cells showing immunopositive expression of CD138 IHC - Immunohistochemical; CD138 - Cluster of Differentiation 138

The patient was diagnosed with small bowel obstruction secondary to an ileal mass. Given that standard endoscopy could not reach the mass, the decision was made to proceed with an exploratory laparotomy. During the laparotomy, a fist-size mass was identified and removed 3 feet from the ileocecal valve. After the mass was resected, the specimen was sent for histopathologic analysis. Within three days, the patient's symptoms improved; he could tolerate solid food and started having bowel movements. As a result, he was discharged from the hospital. Eventually, the surgical pathology report showed an 8.5 cm tumor involving the full thickness of the bowel wall from mucosa to the serosa with vast areas of necrosis, acute inflammation, and abscess formation (Figure 2). The tumor cells were round, discohesive, and many displayed a prominent central nucleolus and pink cytoplasmic globules morphologically consistent with Russell and Dutcher bodies (Figure 3). IHC analysis was performed, and the tumor cells showed positive expression of CD138, EMA, weak BCL-2, and lambda light chains (Figure 4). They showed nonspecific staining for kappa light chains.

The absence of anemia, hypercalcemia, renal dysfunction, or bone pain made MM unlikely as a diagnosis. However, further workup was still recommended to establish the diagnosis of SEP. The workup included serum and urine protein electrophoresis with immunofixation, serum kappa and lambda free light chain (FLC) measurement, and BM biopsy.

No monoclonal protein was detected in the serum or 24-hour urine protein electrophoresis. Analysis for free kappa and lambda light chains was unremarkable. He also underwent an 18-Fluorodeoxyglucose positron emission tomography (FDG-PET) scan, which was unremarkable with no pathologic findings. BM biopsy showed normocellular marrow with trilineage hematopoiesis with no morphologic, immunohistochemical, or flow cytometric evidence of plasma cell neoplasm involvement. MM panel by FISH of the BM sample showed normal results with no evidence of gain of CKS1B at 1q, t(4;14), t(11;14), t(14;16), t(14;20), gain of chromosome 9 or 11, or TP53 deletion. 

The final diagnosis of SEP was established, and the patient's case was reviewed during a tumor board conference. Based on the clear resection margins and lack of lymph node involvement, the consensus was not to offer adjuvant therapy and to proceed with surveillance with repeat serum and urine protein electrophoresis with immunofixation, kappa and lambda FLC measurement, complete blood count, CMP, and CT of the chest, abdomen, and pelvis in 6 months. 

**Figure 5:**
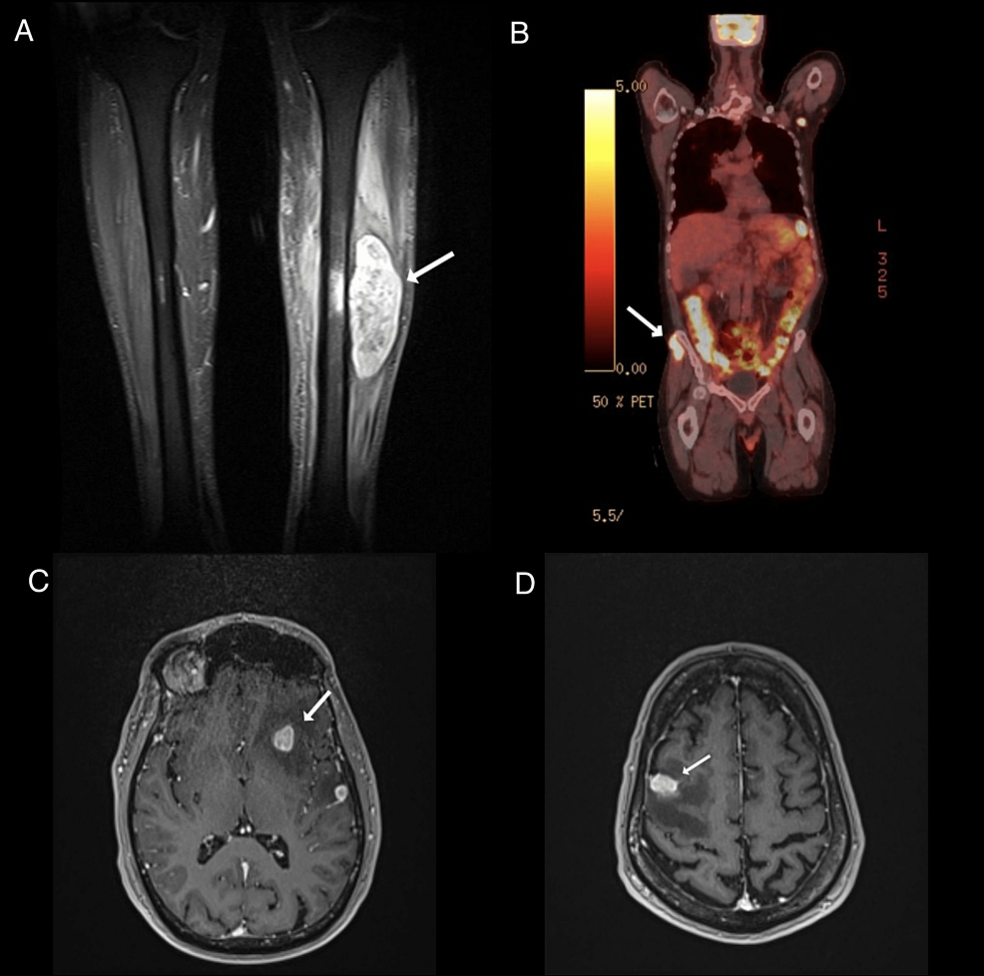
MRI of the left leg showing Intramuscular mass in the tibialis anterior muscle (A). FDG-PET scan showing 2.6 cm hypermetabolic soft tissue mass in the subcutaneous fat and gluteus medius along the right iliac crest (B). MRI of the brain shows enhancing lesions in the brain's left (C) and right (D) hemispheres. MRI - magnetic resonance imaging; FDG-PET - 18-Fluorodeoxyglucose positron emission tomography

Approximately eight months after the diagnosis of SEP, the patient started experiencing left leg pain. A magnetic resonance imaging (MRI) of the left tibia and fibula was performed, which showed a large centrally necrotic intramuscular mass in the tibialis anterior muscle (Figure 5). The mass was biopsied, and pathology showed ALK-negative T-cell anaplastic large-cell lymphoma. Further evaluation with an FDG-PET scan was done, which revealed a 2.6 cm hypermetabolic soft tissue mass in the subcutaneous fat and gluteus medius along the right iliac crest, numerous hypermetabolic lymph nodes throughout the neck, chest, and pelvis, and multiple hypermetabolic pulmonary nodules (Figure 5). The patient was diagnosed with stage IV T-cell anaplastic large-cell lymphoma. After discussing therapy options, he chose not to undergo any treatment. Seven months later, he developed seizures and underwent an MRI of the brain, which revealed the presence of at least five enhancing lesions in the brain (Figure 5). These findings were consistent with metastatic disease in the brain. The patient's condition deteriorated quickly, and a few days later, he passed away. This occurred approximately 15 months after the initial diagnosis of SEP.

## Discussion

Solitary plasmacytomas (SP) are single localized neoplasms consisting of monoclonal plasma cells characterized by the absence of bone marrow plasmacytosis. Based on their location, they can be categorized into two types: solitary bone plasmacytomas (SBP), which are tumors involving the bones, and solitary extramedullary plasmacytomas (SEP), which are tumors that arise in soft tissues [1]. SP is further differentiated into SP with no BM involvement and SP with minimal BM involvement, which is characterized by the presence of less than 10% plasma cells on BM biopsy [5]. 

SP is a very rare condition, occurring at a rate of only 0.34 per 100,000 people per year. The incidences of SEP and SBP are at 0.10 and 0.15 per 100,000 people, respectively [2]. The most common sites for SEP are the head and neck, as well as the upper respiratory tract [1,5]. Only 4% of SEP is found in the GI tract [3]. Interestingly, although the rate of transformation to MM at two years is higher with SBP when compared to SEP, at 37% and 7%, respectively, the relative survival rate at two years is lower for SEP than for SBP, at 77% and 90%, respectively [2]. These findings highlight the complexity and variability of these diseases. Imaging studies such as CT, MRI, and FDG-PET scans are used to accurately identify and characterize the bone lesions or soft tissue masses, as well as to exclude the presence of other lesions in sites different from the primary [1]. SEP is diagnosed based on the following criteria, which include: a single soft tissue tumor with the presence of monoclonal plasma cells on biopsy of the mass, absence of additional tumors on imaging, no bone involvement, absence or minimal (<10%) BM plasma cells, no clinical or laboratory features of MM such as hypercalcemia, renal dysfunction, anemia, and bone pain [1,2,5]. Definitive diagnosis is made with a biopsy of the tissue with histological evaluation, which typically shows the presence of homogeneous infiltrate of monoclonal plasma cells and IHC analysis, which shows an expression of consistent markers such as CD138 and MUM1/IRF4, and is also often positive for CD38, CD79a, CD56, CD117, EMP, and CD27 [1,3,4]. Monoclonal light chain restriction seen on flow cytometry or by PCR can be used as proof of monoclonality [1,3]. FISH is a method of cytogenetic analysis that is used to evaluate for genetic abnormalities frequently associated with SP, such as IGH rearrangement and chromosome 13 loss. In cases of SP, FISH is negative for MALT1 and PAX5 rearrangements [1,4]. BM biopsy with IHC analysis is necessary to evaluate the extent of plasma cell infiltration of the BM to exclude MM [1]. SEP in the GI tract commonly presents as abdominal pain, bowel obstruction, GI bleeding, intussusception, perforation, and fistula formation, depending on the location of the lesion [3,6]. The stomach is the most frequently affected site within the GI tract. Occurrences of SEP in the jejunum are extremely rare. [6]. The diagnostic process for GI SEP usually begins with imaging studies, such as CT or FDG-PET scan, which can be used for diagnosis and for monitoring of disease progression and the response to treatment [3]. The next step in the diagnostic process would be an upper or lower GI endoscopy or, if clinically more appropriate, an exploratory laparoscopy [3]. When a mass is identified in the GI tract, the differential diagnosis remains broad and includes angiosarcoma, adenocarcinoma, gastrointestinal stromal tumor, lymphoma, sarcoma, and others [3]. The definitive diagnosis is made by a pathologic examination of the tumor. The specimen can be obtained by endoscopic biopsy with or without ultrasound guidance or by resection during laparoscopy [3]. SP has been shown to have high response rates when treated with radiation therapy, with patients who received radiotherapy having a 12% relapse rate compared to a 60% relapse rate in patients who did not [1]. The effectiveness of SP treatment is based on retrospective studies with no clinical trials reported at this time. Current data suggest that four weeks of radiotherapy for a total dose of 40-50 Gy has led to significant improvement in outcomes [1]. Radiotherapy alone has been shown to lead to local control in 80-90% of cases. It was also noted that with radiation alone, the rate of progression to MM at five years was about 56% for SBP and 30% for SEP [5]. Surgical management is not always necessary due to the high effectiveness of radiotherapy and is usually used only in cases with symptomatic mass effects from the lesion [5]. Surgery is sometimes used in the initial evaluation phase with total or partial excision of the mass for pathological evaluation. However, even if total excision is performed, radiotherapy is still recommended after surgery to prevent local recurrence [1]. Currently, there is limited and controversial data on the effectiveness of systemic chemotherapy for the management of SP, and no clear recommendations have been made. Further studies are required to draw definitive conclusions [1,5].

Treatment options for GI SEP are similar to the management of SP in other sites and include radiation therapy, surgical resection, chemotherapy, or a combination of them [3]. Chemotherapy medications such as thalidomide, cyclophosphamide, bortezomib, and high-dose steroids have been used in the past in different combinations; however, there is no clear evidence of specific regimens being more potent than others [3]. 

Response assessment is performed by using the International Myeloma Working Group (IMWG) criteria and Response Evaluation Criteria in Solid Tumors (RECIST), which involves monitoring of serum and urine protein electrophoresis with immunofixation, FLC levels, and serum quantitative immunoglobulins every three months for the first two years, as well as FDG-PET scan at three months after radiation and then every 12 months [1,5]. 

As far as we know, there is currently no evidence to suggest an association or shared genetic predisposition between SEP and T-cell anaplastic large-cell lymphoma. However, the genetic abnormalities observed in the two diseases show certain similarities and overlaps, making an association between the two conditions and a transformation from one condition to the other plausible. Therefore, further research is needed to investigate this potential association and shed light on the underlying pathogenic mechanisms. A better understanding of the potential relationship between these two conditions may help improve the management of patients with these diagnoses.

## Conclusions

Our primary objective is to contribute to the existing literature by presenting the case of a patient diagnosed with SEP who underwent surgical resection without additional radiotherapy or chemotherapy. During the observation stage of management, the patient developed T-cell anaplastic large-cell lymphoma. We recognize the limited data available on the optimal treatment strategies for this rare disease and its association with T-cell anaplastic large-cell lymphoma. Therefore, we hope our case will raise awareness and encourage further investigation to improve our understanding of management options for patients with this condition.
